# MOSBIE: a tool for comparison and analysis of rule-based biochemical models

**DOI:** 10.1186/1471-2105-15-316

**Published:** 2014-09-25

**Authors:** John E Wenskovitch, Leonard A Harris, Jose-Juan Tapia, James R Faeder, G Elisabeta Marai

**Affiliations:** Department of Computer Science, Allegheny College, 16335 Meadville, PA USA; Department of Cancer Biology, Vanderbilt University School of Medicine, 37232 Nashville, TN USA; Department of Computational and Systems Biology, University of Pittsburgh, 15260 Pittsburgh, USA; Electronic Visualization Lab, Department of Computer Science, University of Illinois at Chicago, 60607 Chicago, USA

**Keywords:** Visualization, Visual computing, Rule-based modeling, Cell signaling

## Abstract

**Background:**

Mechanistic models that describe the dynamical behaviors of biochemical systems are common in computational systems biology, especially in the realm of cellular signaling. The development of families of such models, either by a single research group or by different groups working within the same area, presents significant challenges that range from identifying structural similarities and differences between models to understanding how these differences affect system dynamics.

**Results:**

We present the development and features of an interactive model exploration system, MOSBIE, which provides utilities for identifying similarities and differences between models within a family. Models are clustered using a custom similarity metric, and a visual interface is provided that allows a researcher to interactively compare the structures of pairs of models as well as view simulation results.

**Conclusions:**

We illustrate the usefulness of MOSBIE via two case studies in the cell signaling domain. We also present feedback provided by domain experts and discuss the benefits, as well as the limitations, of the approach.

**Electronic supplementary material:**

The online version of this article (doi:10.1186/1471-2105-15-316) contains supplementary material, which is available to authorized users.

## Introduction

Modeling approaches used in computational systems biology range from phenomenological to detailed-mechanistic [1]. A popular type of mechanistic modeling uses chemical kinetics, where models are defined in terms of collections of *species* that interact via *reactions* [2]. A shortcoming of the traditional chemical kinetics approach is that the number of distinct species and reactions in a biochemical system can be combinatorially large [3, 4]. A modeling approach that aims to overcome this “combinatorial explosion” is *rule-based modeling* (RBM) [5]. Rule-based models differ from traditional chemical kinetics models in that they explicitly specify the parts of biological molecules that directly participate in and are modified by biochemical interactions [6]. A detailed tutorial on RBM can be found in Ref. [7].

When constructing a model of a biological process, a researcher may begin with a commonly accepted model of the process and build on it over time, modifying and expanding its structure to test different hypotheses. These models can be represented as graphs, with enzymes and other reactants inside cells shown as nodes, and the reaction rules that govern their interactions depicted as edges. As the researcher develops the model, they may make several unrelated alterations, e.g., adding or deleting an interaction in one case while changing the initial concentration of a chemical species in another. These branches in the development of a model can then lead to even more branches. At some point, keeping track of the numerous paths taken in the process of building the model can become unmanageable, with little or no documentation as to how the development of one path was affected by another. Additionally, two models may involve the same molecules with different component structure and interactions, leading to different outcomes. It would be useful for a researcher to be able to directly compare these models, looking for both similarities and differences in their structure.

Although several software tools have been developed for interactive visualization of rule-based models, including RuleBender [8–10], rxncon [11], and Simmune NetworkViewer [12], these tools aim to assist in viewing and understanding one model at a time and do not directly support model comparison, the focus of the current work. As we discuss in more detail below, the problem of model comparison is closely related to that of graph comparison, from which several useful techniques can be adapted.

In this paper, we present MOSBIE (MOdel Simulation Browser and Interactive Explorer), an interactive exploration system that supports pairwise comparison of rule-based models both in terms of model structure and dynamical behavior.

Structural comparisons are performed on the basis of a compact, scalable, visual abstraction called an *interactive contact map* [10, 13]. We define a similarity metric over this contact map abstraction that enables clustering of similar models. Using the map representations and the similarity metric, we then design a visual interface for structurally exploring pairwise differences and family relationships. The utility of the tool is illustrated through two case studies and feedback from domain experts.

## Background

### Task analysis

There are two broad motivations for comparing the similarities and differences within a family of models. In the first case, a research team is building a family of models up from a base model over time. As members leave the project, new members join to replace them. The continuity of the project is thus greatly facilitated by the ability of the new members to browse the history of the model and identify when and where modifications were made. Identifying the common core among the family of models is essential, since the elements that are not present in the core represent modifications to the model.

In the second case, a researcher intends to model a particular signaling pathway or set of pathways. As part of this process, they would want to see what elements of that pathway have been previously modeled, and explore the relationships among existing models in the literature. The researcher downloads several models from one of the several existing online databases [14–17] in a commonly-used model exchange format such as the Systems Biology Markup Language (SBML) [18]. The researcher would like to see at a glance which model components are shared and which are unique.

Starting from these two motivating cases, and through close interaction with domain experts, we identified the following major tasks where visualizations can benefit model comparison in the area of cell signaling. Because of the similarities between model usage in this domain and in other domains, we assert that many of these tasks have global applications to model comparison beyond the cell signaling domain.

1. *Identify similar structures within models*. Identifying similar structures is beneficial because if two different models share a common core, it is likely that those models can be combined to form a single, more-complete model. Additionally, searching for a single structure common to a significant subset of a family of models can help to identify models missing this structure. This can help researchers make observations about the functionality of that subset of models.
2. *Identify structures that differ between pairs of models*. Performing a pairwise comparison similar to task 1 with the goal of identifying structures that differ between the models helps researchers identify model components present in one model that do not appear in the other. Researchers can use this information to explore the functional effects of the structural differences between models. When identifying both the similarities and differences between graphs, minimizing layout differences is essential to enable the user to see changes [19, 20].
3. *Sort/cluster models by similarity*. Sorting models by degree of similarity helps to minimize visual differences between graphs in proximity to each other, facilitating comparison [19]. As such, a method for computing the similarity of a pair of models should be developed or found from literature. Following this, the models should be laid out based on these scores in a clear and visually pleasing way.
4. *Support pairwise detailed comparison*. Building upon the similarity and difference comparison of a pair of models, a researcher should also be able to examine the similar or differing structures of the models in more detail. In particular, the researcher may wish to examine the individual rules within the model to determine the level of similarity.
5. *Explore the functional effects of differences between model structures*. The researcher may also wish to explore the functional effects of model changes. In particular, the researcher should be able to perform a pairwise comparison of the simulation results or other species and reactions in the generated network of a model, in order to identify how the changes within a model affect the generated outputs.
6. *Organize and browse model repositories*. A researcher should be able to use this system to organize and browse a set of possibly unrelated models from a database or online repository. The researcher should still be able to look at the similar and different structures across the collection of models under examination.
7. *Enable the ability to share model layouts withother researchers*. Finally, if a researcher wishes to highlight important structural features that were custom-encoded into a model, that researcher must be able to also convey the structure of the model along with the model itself. To keep the model interactive and to share all of the properties of the model, simply sharing a screenshot of a model is not sufficient. Therefore, although the model language may not specify any kind of set structural information, that structural information needs to be maintained.

This task analysis breakdown shows that a number of problems related to the comparison of models can be solved or aided with visualization. Specifically, Tasks 1–6 can be performed with a clear visual representation of the model(s), and are specifically addressed in this work. Task 7, on the other hand, is not specifically a visualization challenge, but can be facilitated by specific aspects of our visualization system.

### Related work

#### Computing graph similarity

A number of methods have been proposed for computing the similarity of two graphs. Zeng et al. [21] computes a similarity score for a pair of graphs by computing the edit distance between two graphs, counting the number of edit operations to nodes and edges required to transform graph *G* into graph *H*. Bunke and Shearer [22] computes a similarity score by finding the maximal common subgraph, looking for the largest isomorphic subgraph present in graphs *G* and *H*. Ullman [23] presents an algorithm to find subgraph isomorphisms using a brute-force tree search, but pruning the tree to reduce the number of successor nodes that need to be examined. Our approach for determining a graph similarity score builds off of these ideas, looking both at maximal common subgraphs, while also considering the differences between graphs that can be computed through edit counts.

#### Simulation journaling

A number of recent projects have included simulation journaling components to track simulations, steer computations, and perfect models. The World Lines system [24–26] simulates flood response and control, storing options for different simulations as a timeline tree. Similar to World Lines are tracking graphs, as seen in Widanagamaachchi et al. [27], which show the evolution of features over time as a collection of feature tracks that may merge or split. Likewise, the PORGY system [28] enables simulation steering through direct manipulation of graph components, using a node-link representation to show transitions between graph states. Our system notes the links between models and simulations through their proximity to each other as computed by the custom similarity score noted above.

#### Visualizing temporal network changes

Misue et al. [19] notes that the primary factor to consider when visualizing network changes over time is preserving the user’s mental map — minimizing unnecessary changes to the structure of the graph while emphasizing patterns within the graph. Four primary mechanisms have been used for visualizing changes in networks while preserving the mental map. Using an extra dimension to show network changes over time can add to clutter in the visualization, but can be effective when used appropriately, with either a full extra dimension [29] or simply a “half-dimension” [30]. Small multiples are useful to show side-by-side comparisons of two or more networks, but with the disadvantage of losing some of the detail of the networks due to the reduced size, and are featured in ego networks [31] and the Semantic Graph Visualizer project [32]. Animations are useful in directly showing how a graph transforms over time, but have the occasional issues of being overly complex or too fast to be accurately perceived [33]. Such animations have been studied in projects such as DynaVis [34]. Finally, interactions for comparing graph states over time come in various forms, including interactive tree layouts [35], configurable layout algorithms in 3D graphs [36], and time sliders [24].

## Methods

The top design of our tool is informed by our formal task analysis (see “Background”). Since many of these tasks feature comparisons, we selected a small multiples top design; this design allows the comparative exploration of models. We mitigated the issue of detail loss by providing a zoom function to the individual multiples, using animated transitions in the zoom action as suggested by Shanmugasundaram and Irani [37] and using slow-in/slow-out pacing as recommended by Dragicevic et al. [38]. The front-end also allows the exploration of previous simulations and versions for a specific model.

The small multiples view provides a compact, scalable, visual encoding of models through an abstraction called an *interactive contact map* [10, 13]. The view further allows the comparison of similarities and differences between pairs of models represented as contact maps. An interactive contact map is a compact, interactive graph representation of a complete model [10]; this representation lies at the core of our scalable approach. The molecules and binding sites in the biological model become nodes in an undirected graph, while the reaction rules are mapped to edges and component states. The contact map provides a global, compact view of the model. As discussed below, the interactive contact map can visually map models featuring hundreds of species and thousands of reactions into compact graphs featuring dozens of edges and nodes. The small multiples view is enabled by three modules: a Contact Map Manager, a Comparison Engine, and a Layout Stabilization and Overlay Module.

### Contact map manager

The Contact Map Manager handles the parallel loading of a family of models from disk, generating contact map representations for these models and laying the contact maps out on screen. It further supports interactions such as panning and zooming, highlighting similarities and differences between pairs of models, identifying common edges and nodes across an entire family of models, showing the states of a model, and opening the model in the default editor interface for closer inspection of parameters and simulation outputs.

**Figure 1:**
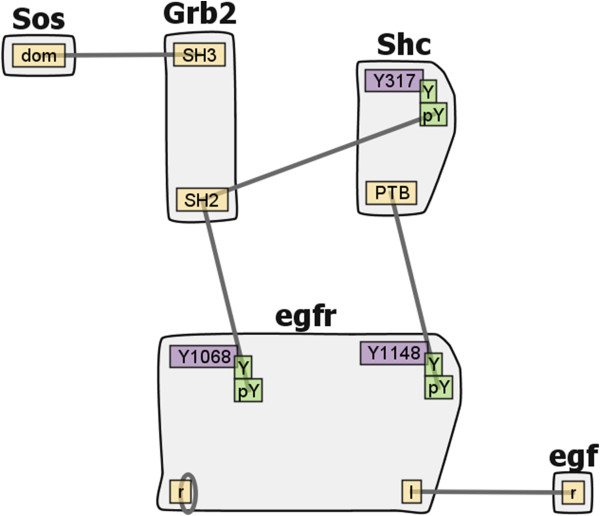
**EGFR Contact Map.** An example contact map from the *EGFR* family. The model contains five distinct types of molecules (shown as grey aggregates), each comprised of one or more components (yellow and purple rectangles), which can have one or more states (green rectangles). Each edge represents a possible binding interaction to a component (possibly in a given state), specified by one or more rules. Components shown in purple have states that are modified by the action of one or more rules. The rule-based model represented by this contact map generates a reaction network with 356 species and 3,749 reactions.

Each contact map is kept concise and scalable by limiting the number of nodes in the map: molecules and binding sites are uniquely identified by single nodes, regardless of how many times they appear in the model rules. For example, the *epidermal growth factor receptor* (*EGFR*) model in Figure 1 contains 24 different reaction rules, which are compressed into a contact map with six edges and three modifiable components. The rules of the model generate a system of 356 species and 3,749 unidirectional reactions involving those species. It is worth noting that the contact map is an abstraction of the generative-model for the system, not of the implied reaction network. Molecules are represented as large nodes that contain smaller, internal nodes that represent binding sites (yellow nodes) and components containing states that are modified by the rules (purple nodes). Note that a modifiable component may also participate in bonds (e.g., the *Y317* component of *Shc*). The reaction rules in the model are represented as the edges connecting the binding sites, and several rules may map to a single edge.

By default, the contact map is drawn using a force-directed layout algorithm which is intended to minimize edge crossings to preserve clarity. A user can manipulate the location of the nodes to convey structural information about molecules and components with the layout of the graph [10] (discussed further in “Layout Stabilization”). The contact maps corresponding to the members of a model family are laid out in a small multiple display and rendered in grayscale in order to focus attention on the similarity and difference highlights generated by the Comparison Engine.

### Comparison engine

The Comparison Engine serves two major purposes. First, it sorts the models by complexity in order to minimize differences between neighboring panels and thereby preserve the viewer’s mental map of the core model. Second, it calculates similarities and differences between the models, both pairwise and across the full family, which are then passed to the Contact Map Manager for display.

#### Sorting models

**Figure 2:**
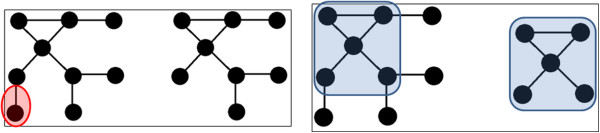
**Model Overlaps.** (Left) Two graphs that share a large number of nodes and edges. The only difference between these two graphs is the red highlighted node and edge. (Right) Two graphs that share a common structure. The blue highlighted region has an identical structure, and so the second graph is a subgraph of the first.

To compute the visual similarity of a graph (i.e., contact map), we create first an adjacency matrix representation. In an adjacency matrix, each row and column is labeled with a node from the graph, and the matrix itself contains a 0 or 1 depending on whether or not an edge exists between the two nodes. In our contact map implementation, a node can either be a molecule, a component, or a state. We follow a bottom-up approach in the construction of the adjacency matrix. Starting from the finest granularity, a state is guaranteed to be a row/column in the adjacency matrix. A component is included as a row/column if it has no states already included in the matrix. A molecule is guaranteed to have at least one component, although that component could represent the entire molecule. This numerical representation of the graph enables us to construct a visual similarity metric as described below.The first challenge in computing a similarity score for two models is defining what makes two graphs similar. There are two descriptive examples for model pairs that are similar, shown in Figure 2. In the first, two graphs share a large number of nodes and edges, representing a majority of each graph. These two graphs are certainly similar, as their only differences represent a small percentage of the overall structure. In the second example, two graphs only share a small number of nodes and edges, but one graph is a subgraph of the second. Since the structure of the smaller graph is mostly (or completely) contained within the larger graph, we can argue that these graphs are also similar.

To account for both of these similarity examples, we propose and construct four similarity matrices which reflect graph similarity. A similarity matrix has the same basic structure as an adjacency matrix. However, instead of nodes in the rows/columns, a similarity matrix contains an entire model. Instead of Boolean edge existence values inside the matrix, a similarity matrix contains a real number representing how similar two graphs are by some measure.

The first two of our similarity matrices handle the first similarity example case. One similarity matrix counts the number of nodes that the two graphs share, while another counts the number of edges that the two graphs share. The other two similarity matrices handle the second similarity example case. One similarity matrix counts the percentage of nodes that the two graphs share, while another counts the percentage of edges that the two graphs share. In each of these cases, we calculate the percentage of nodes/edges in the smaller graph that are present in the larger graph. Hence, if graph *G* is a subgraph of graph *H*, then the similarity score by this measure is 100% for both nodes and edges, regardless of the number of nodes in graphs *G* and *H*.

We calculate an absolute similarity score by multiplying the number of nodes that the graphs share and the percentage of nodes that the graphs share, multiplying the number of edges that the graphs share and the percentage of edges that the graphs share, and finally adding these two values together. Thus, to compute the absolute similarity between two graphs, we use of the following similarity formula:
1

**Figure 3:**
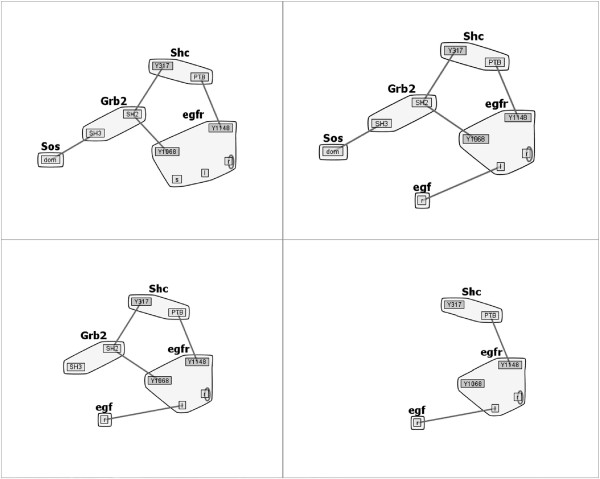
***EGFR*** **Models.** Four sample multiples from the *EGFR* family of models sorted by similarity score relative to the most complete model (upper left). The most complete model generates a reaction network with 356 species and 3,749 reactions.

To sort the models in our small multiples view, we precompute similarity scores for each pair of graphs. We also compare each graph to the *most complete graph*, and sort the models row-wise into the small multiples view based on their similarity score in comparison with the most complete model. In our implementation, we assume that the most complete graph is the graph with the greatest number of nodes and edges. The assumption is based on our understanding of the iterative development of biological model families - researchers continue to add molecular structures and interaction rules to models to obtain an increasingly complete representation of the physical process. As such, the number of nodes and edges will generally increase as the model is developed. An example of this layout is shown in Figure 3, and is described more fully in “Layout Stabilization and Overlay Module”.

#### Model comparison

The second feature of the comparison engine is its ability to locate similarities and differences between models, which are then displayed using a bubbleset overlay [39]. For computing similarities, we iterate over all nodes and edges in one model, creating an identifier for each. We create a unique identifier for the structure we are searching for (by molecule name, component name, state name, and the number of times seen). We then iterate across all models, searching for that structure (synonyms are currently not allowed). If the structure exists in the other model, we add that structure to an internal list. Once the iteration is complete, the internal list of structures is passed to the Contact Map Manager for display. Similarly, for computing differences, we must iterate over the nodes and edges of both models, looking for structures that exist in one model but not the other. When such a structure is found, it is likewise added to an internal list. In addition to pairwise comparison, our system also supports identifying a single node or edge across the entire model family. This will allow a researcher to identify which members of a family of models contain a certain problematic rule, or a binding site that is no longer of functional relevance to the behavior of the model. Because each of these comparison processes require nested iteration over the node and edge sets of both models, the computational complexity of our comparison algorithm is O(*n*^2^).

### Layout stabilization and overlay module

Laying out the contact maps in a consistent manner across all of the small multiples facilitates the visual comparison of similar models, and in fact may be required for visual comparison in more complex models. We implement layout stabilization by storing the nodes and their user-assigned positions for a particular graph, and then applying the stored layout across a family of models. Nodes not present in the stored layout are assigned positions using a force-directed algorithm. By default, layout positions are taken from each individual model. However, the user may override this choice by selecting a stored layout from a drop-down list.

**Figure 4:**
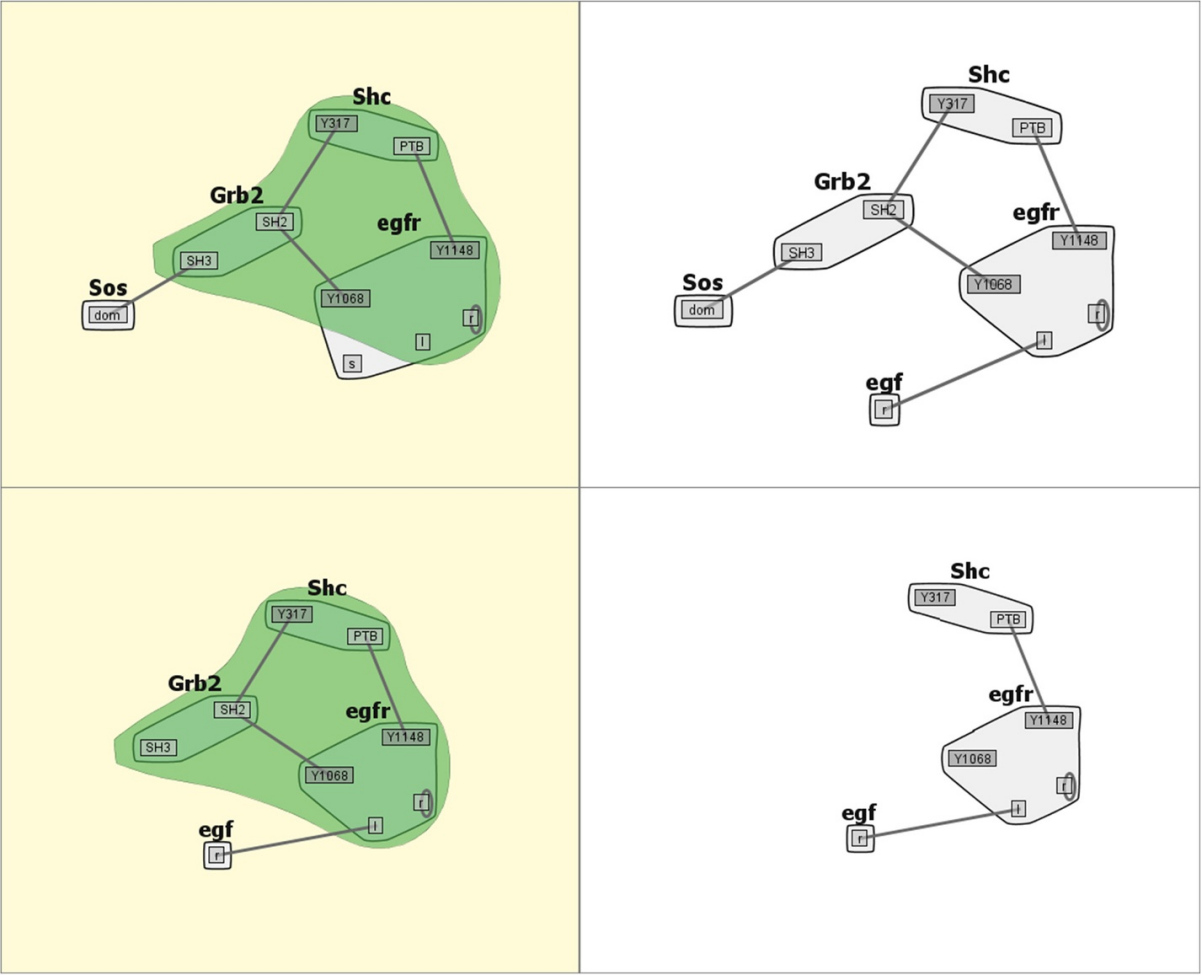
**Similarity Comparison.** The Comparison Engine in action, showing the similar nodes and edges (green highlighted region) between two models from the *EGFR* family. The model (lower left) being compared to the most complete model (upper left) generates a reaction network with 155 species and 1,200 reactions.

When further analyzing a subset of models in a family, it is helpful to easily identify similarities and differences in the structures of each model. The similarities and differences that we compute via the Comparison Engine are highlighted with a bubbleset overlay [39] on the relevant small multiples. When running a differences comparison, if one graph is a complete subgraph of the other, the result in the smaller graph will be an empty overlay bubbleset (an example of this empty bubbleset can be seen in section Case study 1: comparison of a model family below). To show that this subgraph model is a member of the comparison, the backgrounds of models under pairwise comparison are highlighted. An example of a similarity comparison bubbleset highlight with layout stabilization is shown in Figure 4.

Figure 4 emphasizes the scalable advantages of the interactive contact map representation. The system compactly represented here with at most six edges generates over 300 molecular species and more than 3,000 unidirectional reactions among those species. The contact map representation combined with layout stabilization makes similarity and differences between the models easy to spot. For example, in the highlighted panes of Figure 4, it can be seen that the two models share the molecules *Grb2*, *Shc*, and *egfr* but differ in that *egfr* in the top model has an additional component. Also, the top model contains the molecule *Sos*, which can bind *Grb2*, whereas the bottom model does not. However, the bottom model contains *egf*, which can bind *egfr*. Given the size of the rule sets of the underlying models corresponding to each of these multiples, these differences would be arduous to determine from text-based comparison of the rules, or from unprocessed network diagrams of the two systems.

## Availability and requirements

The MOSBIE system is open source and cross platform, with 32- and 64-bit releases available for Windows, Linux, and Mac OS X. The system uses Java, Rich Client Platform (RCP), Perl, and Prefuse libraries. MOSBIE is implemented as a perspective in the RuleBender interface for rule-based modeling [8, 9]; the RuleBender release includes the BioNetGen software [6] as well as NFsim, which is an additional simulator that allows for efficient simulation of large models [40]. No installation is required: unzip the downloaded archive to a directory and the application will run directly. The system can be downloaded at http://visualizlab.org/mosbie. Sample models are located in the SampleModels/BNG directory of the decompressed directory. All the example models and auxiliary layout files used in this paper can also be found in the Additional file 1 provided with the manuscript.

## Results

In this section we report on the performance of MOSBIE. We follow with two case studies from the application domain, and finally report feedback from domain experts.

### Performance analysis

We report the time required to calculate the similarity matrices, as well as the time to sort a collection of models, using an HP Pavilion g7 machine with 6 GB RAM and an i3 2.3 GHz dual-core processor. Our test set of 20 models from the *fceri* family represents biological systems with thousands of species and tens to hundreds of thousands of reactions. For example, the *fceri_fyn* model generates a reaction network of 1,281 species and 15,256 reactions, and the *fceri_fyn_trimer* model generates 20,881 species and 407,308 reactions. In their interactive contact map representations, the models in this family are captured as graphs with between 16 and 21 nodes and 4 to 5 edges.

**Figure 5:**
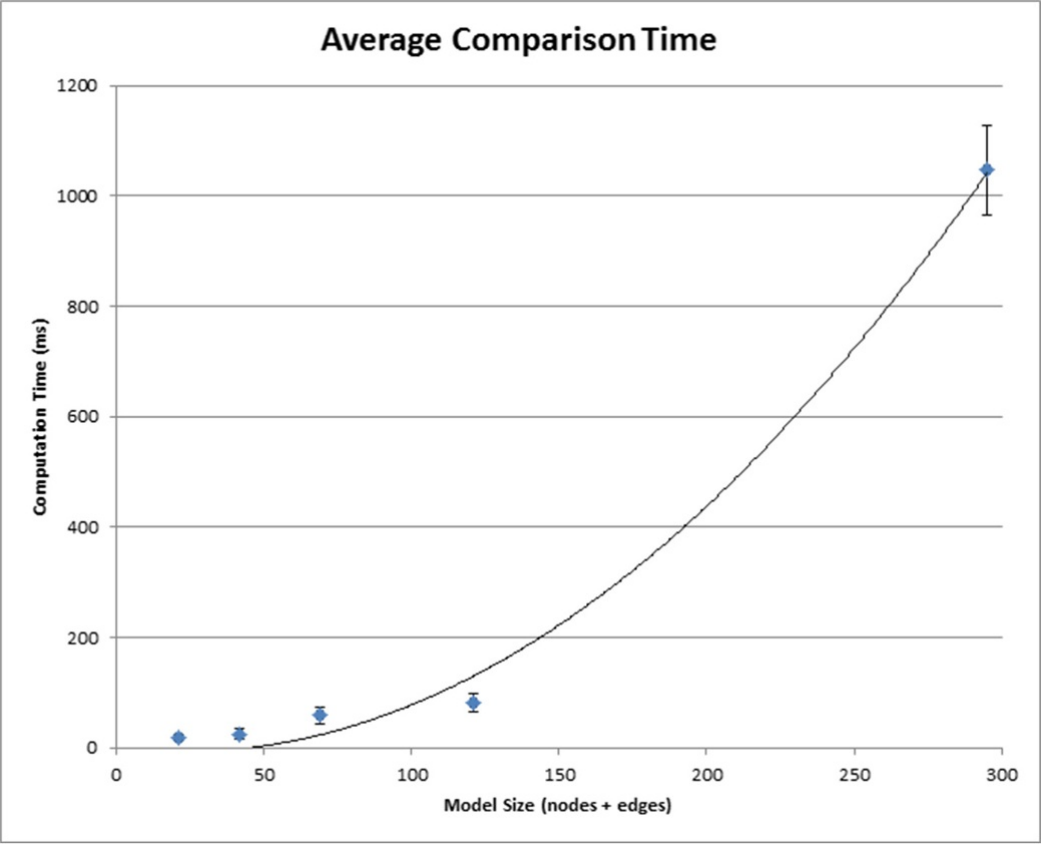
**Comparison Time.** The average calculation time of 10 similarity and difference comparisons on five different model families. The error bars represent one standard deviation in comparison time. The curve of best fit follows a quadratic equation, which follows from the O(*n* ^2^) comparison algorithm (see “Methods” for a complete algorithm description).

The model set includes the nine models reported below in the first case study, plus eleven duplicates of these nine models to reach a total of twenty. This duplicate set construction enables the performance evaluation of our approach on a larger set models of the same significant size as the original *fceri* family. The duplicate approach is reasonable in this case: because it iterates through models in the same fashion regardless of their structure, the comparison algorithm computing time is not reduced when duplicate models are compared. We found that computing the four similarity matrices on this set (see “Sorting models”) required 0.25 seconds and that sorting the models based on their similarity to the most complete model required 0.0052 seconds. This computation time stands in contrast to the 14.56 seconds required to load the collection of models from disk and build the contact maps.To evaluate the performance of our browsing system, we calculated the average computing time for model comparison. We evaluated computing both similarities and differences across five model families, including the three families reported in the case studies and feedback section (Figure 5). Smaller model families ranging from 21 to 121 combined nodes and edges took less than 100 milliseconds for comparison runs. The largest model family we attempted had a combined 295 nodes and edges; the mean comparison time was slightly over one second.

Using the same test set of models and identical machine configuration as in the previous experiment, we computed the amount of time required to (i) locate a node in the family of models, (ii) locate an edge in the family of models, (iii) compare the similarities between a pair of models, and (iv) compare the differences between a pair of models. In all cases, the comparison took less than a quarter of a second to complete, including the call to the Comparison Engine and the display refresh.

### Case study 1: comparison of a model family

In this case study, a computational biologist explores a family of rule-based models that describes signaling through the *Fc εRI* membrane receptor [41], looking at various properties of the set of models. The biologist begins by loading the family of models; a directory is selected through a standard dialog box and all models in that directory are loaded into the system. Reading the models from disk and generating the contact maps from the rules requires roughly one second per model. As the models are loaded, the Comparison Engine computes the similarity matrices for the family, sorts the models, and lays them out appropriately into the small multiples panel. This reflects Task 3 from our task analysis.

**Figure 6:**
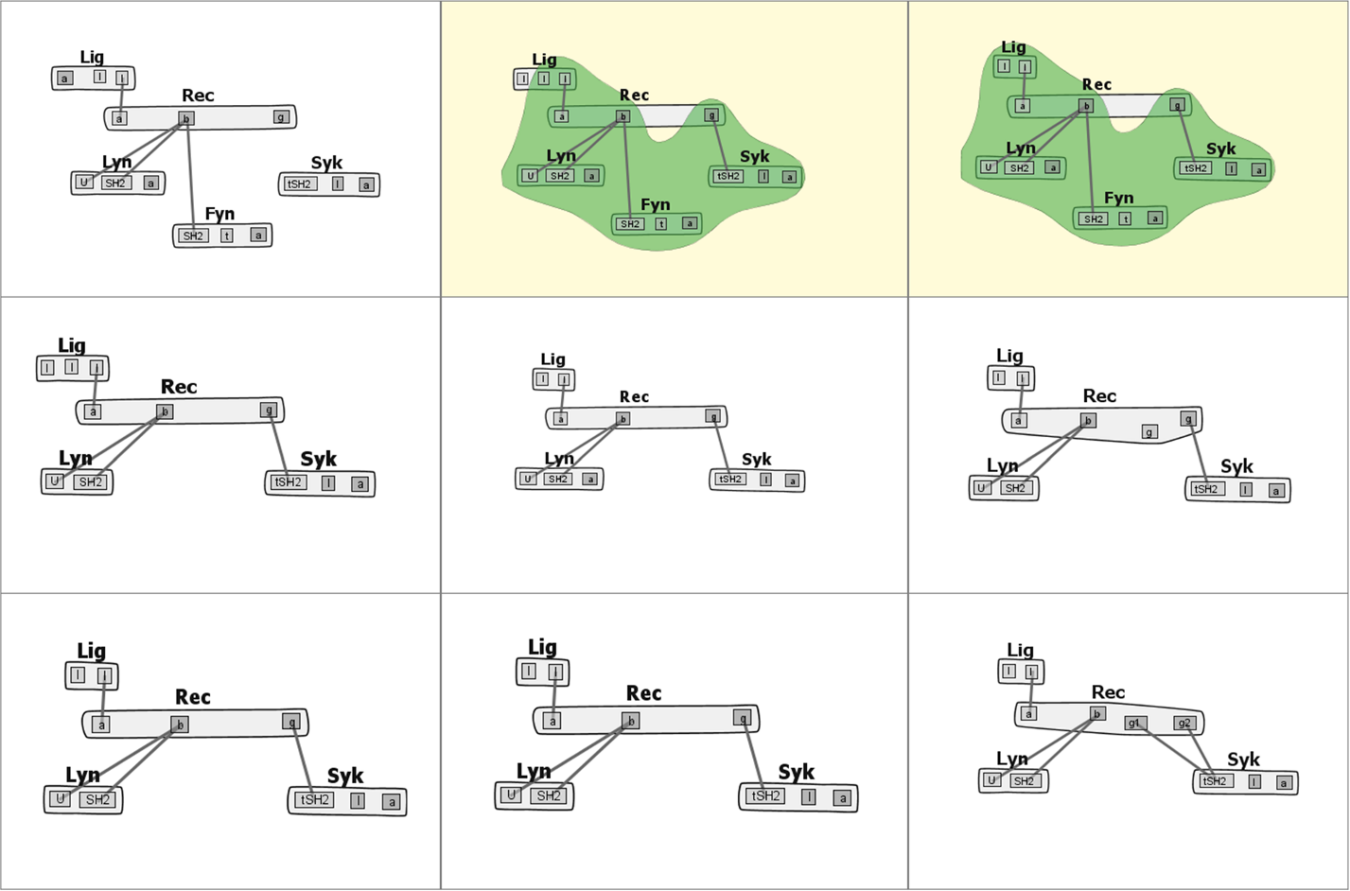
***fceri*** **Similarity Comparison.** A similarities comparison within the *fceri* model family, highlighting the similarities between the *fceri_fyn* and *fceri_fyn_trimer* models. The *fceri_fyn* model (top right) generates a reaction network with 1,281 species and 15,256 reactions, while the *fceri_fyn_trimer* model (top middle) generates a reaction network with 20,881 species and 407,308 reactions.

Next, the biologist enables layout stabilization across the model family (Task 7). With this new layout, the biologist notices that all of the models seem to have a common core structure, with a large *Rec* molecule centrally located, and surrounded by *Syk*, *Lyn*, *Lig*, and occasionally *Fyn* molecules. To confirm that this common structure does indeed exist, the biologist selects the “Compare similarities” radio button, then begins to select pairs of models to compare. Through this selection process (which maps to Task 1), the biologist confirms via a bubbleset overlay that this core structure does exist throughout the model family, with a few small differences. One such comparison is shown in Figure 6.

**Figure 7:**
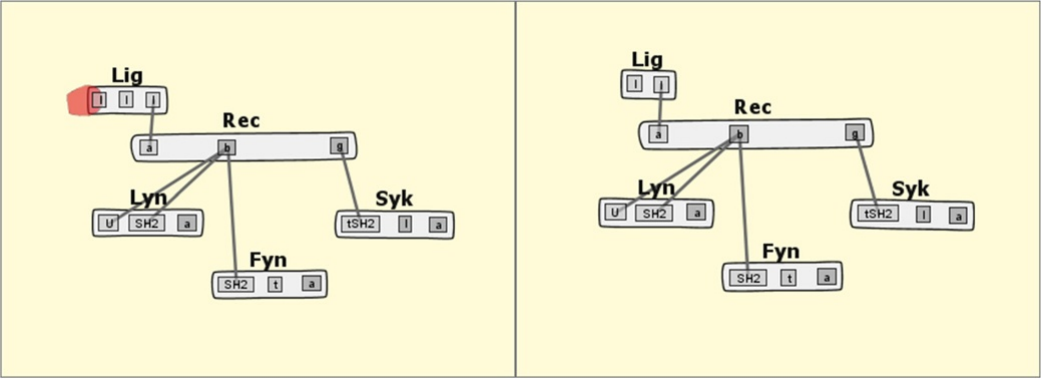
***fceri*** **Differences Comparison.** A differences comparison between the *fceri_fyn_trimer* (left) and *fceri_fyn* (right) models, showing a binding site in *fceri_fyn_trimer* that does not exist in *fceri_fyn*.

**Figure 8:**
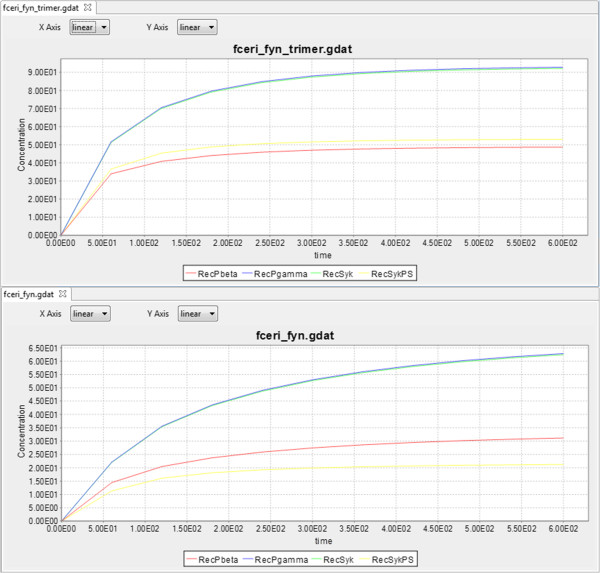
**Simulation Outputs.** The simulation outputs for *fceri_fyn_trimer* and *fceri_fyn*, showing similar curves for the concentrations of *RecPbeta*, *RecPgamma*, *RecSyk*, and *RecSykPS*, but with *fceri_fyn_trimer* having concentrations 50% higher than *fceri_fyn*.

To investigate some of these differences more closely, the biologist switches the radio button selection to “Compare differences,” which generates a different bubbleset overlay. In one case, comparing the *fceri_fyn* and *fceri_fyn_trimer* models, the biologist notices that a single binding site in the *Lig* molecule differs between the two models (Figure 7). This “compare differences” action maps to Task 2 from our task analysis. Noting this difference of a single binding site, the biologist now wishes to learn how this change in the model affects the concentrations of certain species in the model simulations. Even subtle changes to the model can result in significant changes in the network output. By selecting the “Open Simulations” option from a context menu on either highlighted model, the most recent simulations for each model are identified and opened for the researcher to compare. These simulations are shown in Figure 8, which addresses Tasks 4 and 5. It should be noted that to validate the significance of this comparison the user would need to check that the parameter values governing reaction rates and initial species concentrations were the same between the two models. This can be done in several steps in MOSBIE by opening the corresponding model input files and comparing the parameter blocks.

With the simulation outputs displayed, the researcher can note that, while the concentrations of the observables follow similar curves, the *fceri_fyn_trimer* outputs grow at a rate roughly 50% faster than those in the *fceri_fyn* model (Figure 8). Additionally, the concentration of *RecSykPS* is higher than the concentration of *RecPbeta* throughout the full simulation of *fceri_fyn_trimer*, whereas the opposite occurs in *fceri_fyn*. From this observation, the researcher notes that it is clear that the addition of a third ligand site significantly increases the rate of phosphorylation of the receptor (*RecPbeta* and *RecPgamma* curves in Figure 8) and of *Syk* (*RecSykPS* curve). The effect on *Syk* phosphorylation is amplified in comparison to the effect on receptor phosphorylation, which is seen by a change in the ordering of the curves in the top and bottom panels.

It is worth emphasizing that the comparisons shown in Figures 6 and 7 involve large models. The *fceri_fyn* model generates 1,281 species and 15,256 reactions and the *fceri_fyn_trimer* model generates 20,881 species and 407,308 reactions. These models may take several hours to generate and simulate. As noted by the domain experts, MOSBIE reveals structural differences between models based on existing simulation data, without the user having to regenerate the results. Thus, MOSBIE potentially saves hours of simulation time.

### Case study 2: comparison of models from a database

In this second case study a researcher is developing a model of the EGFR signaling network. The researcher wants to see what molecules and interactions have been included in previous models, with an eye toward integrating these into the new model. The researcher finds two models of EGFR signaling in the BioModels database [14], with model IDs BIOMD0000000019 (Model 19) and BIOMD0000000048 (Model 48), and downloads them as reaction networks in SBML format. Both models are fairly large — Model 19 has 87 species and 236 reactions and Model 48 has 23 species and 47 reactions — and the only visual representations of the models provided in the respective papers [42, 43] use different nomenclature and layout, making them difficult to compare visually.

These models are not rule-based and the molecular compositions of the species in each model are not explicitly provided. However, the models can be converted into a rule-based format and the species’ molecular compositions recovered using a recent web-based tool called the Atomizer [44]. Following successful translation to BioNetGen language (BNGL) format, both models are loaded into MOSBIE and their contact maps displayed. Because the two models use slightly different names to refer to some of the molecules they share in common, these mappings had to be identified and modified manually in the model editor.

**Figure 9:**
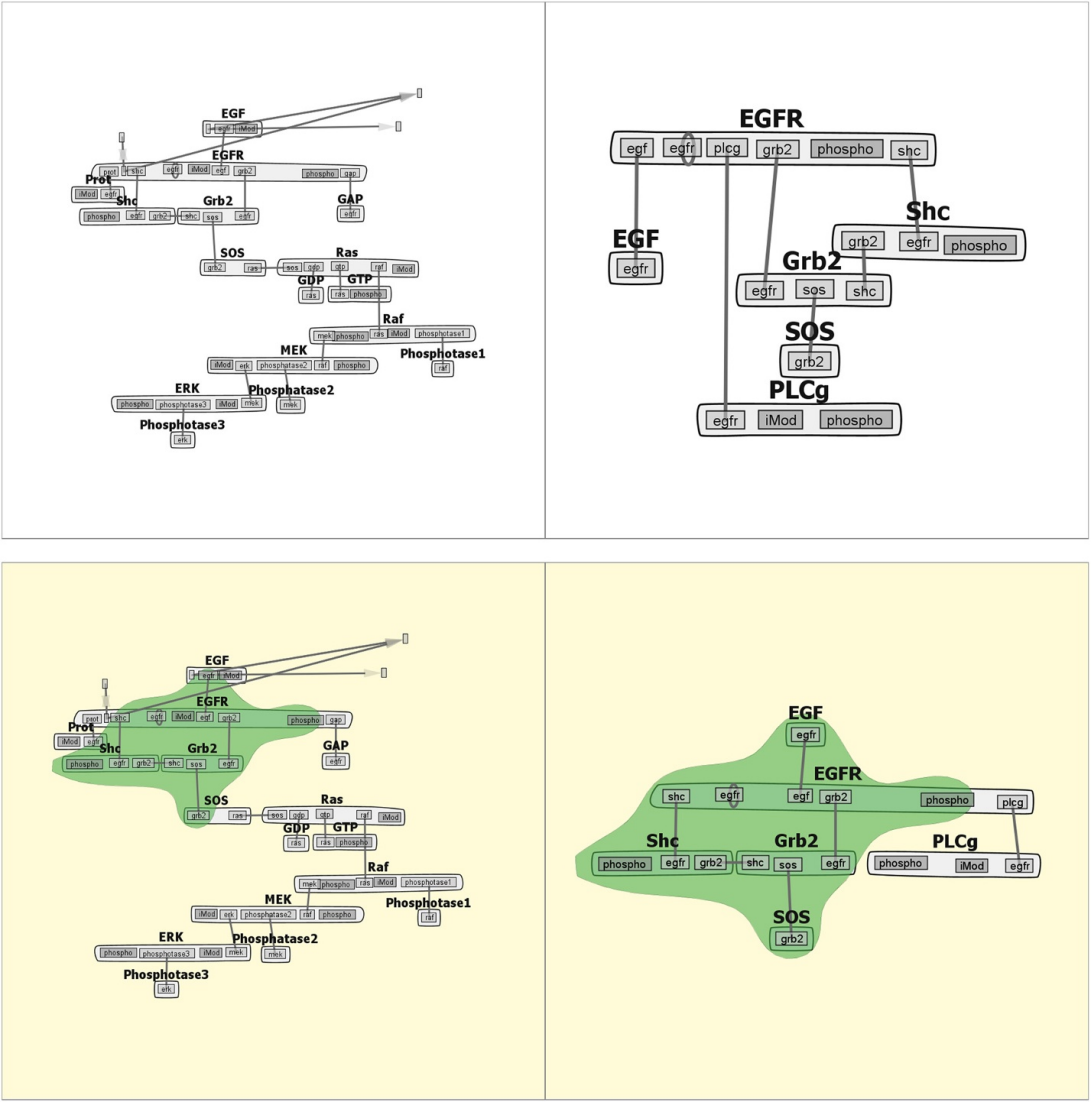
**Comparison of two models of EGFR signaling from the BioModels database** [14]**.** Left column: BIOMD0000000019 (Model 19) [42]; right column: BIOMD0000000048 (Model 48) [43]. Contact maps with custom layout (top row) and similarity comparison with layout stabilization (bottom row) for the two models are shown. The region highlighted in green shows that the two models share a core set of molecules, components, and interactions. Model 19 is comprised of 87 species and 236 reactions, whereas Model 48 is comprised of 23 species and 47 reactions. Note that both models are encoded in SBML and are not rule-based models. Contact map representations were generated by extracting the implicit molecular structures of these models using the Atomizer [44].

Manual layout of the contact maps reveals the implicit molecular components and interactions of the original model (top row of Figure 9). The initial layout of the contact maps for the two models is somewhat different. To facilitate comparison, layout stabilization is applied using the layout for the larger model, followed by correction of the position of the *PLCg* molecule in Model 48 and its corresponding binding site in *EGFR* to line up with other molecules and components in the contact map. Selecting the “compare similarities” radio button, followed by zooming and recentering, results in the view shown in the bottom row of Figure 9.

The similarity comparison immediately highlights a core set of elements common to both models. In fact, Model 19 contains all molecules and interactions present in Model 48, except for the *PLCg* molecule. The comparison in Figure 9 also shows that, in addition to containing a number of additional molecules and interactions, Model 19 also considers synthesis and degradation of *EGF* and *EGFR*, which are represented by the unstructured nodes connecting to those molecules.

The similarity in the core structures of the models was not noted in the paper describing Model 19 [42], even though this model was published after the paper presenting Model 48 [43]. Without MOSBIE, it is difficult to identify similarities and differences between models published in the literature because they are usually presented in the form of long lists of equations that use different nomenclature. Although the nomenclature problem must still be addressed manually, in our opinion this case study demonstrates the power of MOSBIE to enable model comparisons that would be prohibitive without monumental effort.

### Domain expert feedback

**Figure 10:**
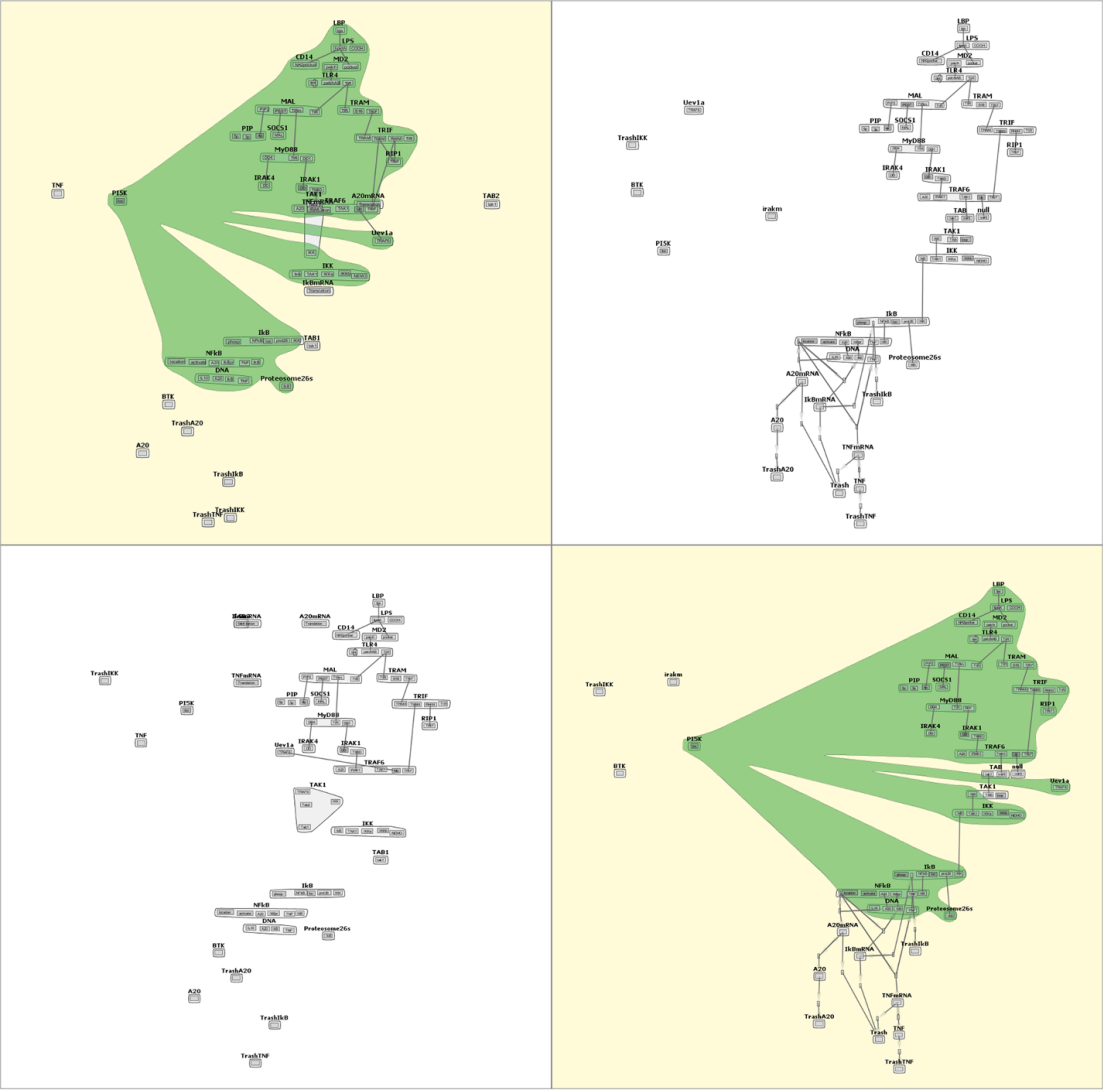
**The TLR4 family of models.** A similarity comparison between models *TLR4_v15* and *TLR4_RPS_v1* in the *TLR4* family. *TLR4_v15* generates a reaction network with 337 species and 2,284 reactions, while *TLR4_RPS_v1* generates a reaction network with 657 species and 3,368 reactions.

In addition to the two case studies reported above, three computational biologist domain experts (co-authors on this work) requested the MOSBIE system for the purpose of exploring model sets. The experts were most interested in using the system for locating core structures that are common across model families, including the *TLR4* family shown in Figure 10. These core structures can be ideal sites for merging similar models into a larger structure.

As noted in “Performance Analysis”, comparison times for a pair of models of the size of those in the *TLR4* family (contact map representations with a maximum combined 295 nodes and edges) approach one second, which is still roughly equivalent to the time required to load each model from disk and generate the contact map. Figure 10 shows a similarity comparison between two models in this family. We found that the researchers were still pleased with this comparison computation time, as it is still significantly faster than a manual comparison.

The domain experts expressed satisfaction with the Layout Stabilization module. They noted that, in addition to making it easier to visually compare models in the explorer view, they could also package the layout information with the model files when sharing models with other researchers. This allows the experts to highlight certain structures in discussions without either providing a screenshot or worrying about differences in the layout computed on each machine. It also enabled the experts to store their own custom layouts for models across multiple sessions.

Discussions with our domain experts led us to develop additional features that were not explicitly presented in the case studies. For example, the experts felt that the ability to highlight the location of a single node or edge across the entire model family (as opposed to the previously mentioned pairwise comparisons) would be useful for identifying which models in a family are missing a key structure. These discussions were also useful for refining some features of the system overall, such as using a grayscale color scheme for the small multiples so that the similarity and difference bubblesets stand out even more.

Finally, our domain experts also praised the ability to browse the results of past simulations, as some model structures result in very large networks that take significant resources to run. In this case, researchers would not want to rerun simulations. Specific to our first case study, the *fceri_fyn_trimer* model requires close to an hour to perform the network generation stage of the simulation.

## Discussion and conclusion

In the absence of a contact map, obtaining a global understanding of the contents of even a single rule-based model from a set of rules in text form is difficult. This difficulty is compounded when doing model comparison. While a binary comparison of two models based on ≲ 30 rules could be done by hand — by someone well versed in reading rules — MOSBIE offers the power to compare many models at once, as shown in the first case study. This first case study, where we compare nine different models with relatively subtle structural differences, illustrates this scaling issue. As shown in both case studies, MOSBIE allows detection of patterns that might otherwise be difficult to see.

The results of our case studies indicate that MOSBIE effectively meets the tasks we have identified for browsing sets of models (Tasks 1–5 in “Task Analysis”) without requiring specialized training on the system. Our domain experts were able to begin to explore the families of models immediately, noticing similarities and differences in model structures and identifying relationships between the model results that were being compared.

Task 6, organizing and browsing online repositories, is not discussed in the case studies because there is currently no such online repository for rule-based models. However, introducing an online database of models is an interesting research direction that we are currently pursuing [45]. When such a repository is developed, our system will be useful for comparing models that overlap in their composition, provided that a consistent annotation scheme is used to allow for accurate determination of common model components.

The visual comparison features that we have implemented could facilitate model merging in situations where models are developed by multiple research groups (Task 7). A prerequisite step for comparison of models developed by different groups is the modification of identifiers — molecule names, component names, and component states — such that shared elements have the same identifiers in all of the models being compared or merged. Differences in protein nomenclature are, however, common in the literature [46]. Annotations such as UniProt ID numbers (http://www.uniprot.org/) could also be employed to facilitate identification of common identifiers. A tool that allows synonyms in the protein nomenclature is an interesting direction of future work. In addition, to fully accomplish cross-group model merging additional interface features would be required, such as visual molecule and rule merging.

A limitation of MOSBIE is that the browser-view model comparison is currently based only on contact maps. It is possible for models with similar but distinct rule sets to yield identical contact map representations in this browser view. Detecting such differences would require performing comparisons on more fine-grained representations of model structure, using for example the interactive approach described in [9]. However, performing comparisons on more fine-grained representations of model structure in MOSBIE is beyond the scope of the current work.

In conclusion, we have introduced a novel, powerful tool for analyzing structures and dynamics within biochemical model families. Our open-source system uses a compact, scalable visual abstraction called an interactive contact map and a similarity metric over this abstraction to enable the clustering of similar models. An intuitive interface further allows researchers to seamlessly compare pairs of models directly, to identify similarities and differences in the structure of models, and to directly compare model simulation outputs. This approach effectively streamlines the analysis of models, both existing and newly created. Domain expert feedback and two case studies highlight the benefits of using this exploratory system in the context of systems biology.

## Electronic supplementary material

Additional file 1: **The BNGL models and position files necessary to generate the figures presented in this work.** (ZIP 57 KB)

## Abbreviations

RBM: Rule-based modeling
MOSBIE: Model simulation browser and interactive explorer
EGFR: Epidermal growth factor receptor
SBML: Systems biology markup language
RCP: Rich client platform
Y317: Tyrosine 317
Shc: Src homology 2 domain-containing-transforming protein C1
Fc *ε*RI: High affinity immunoglobulin epsilon receptor
Rec: Receptor
Syk: Spleen tyrosine kinase
Lyn: Tyrosine-protein kinase Lyn
Lig: Ligand
Fyn: Tyrosine-protein kinase Fyn
RecSykPS: Syk-phosphorylated Syk-receptor complex
RecPbeta: Beta-subunit phosphorylated receptor
RecPgamma: Gamma-subunit phosphorylated receptor
PLCg: Phospholipase C gamma
TLR4: Toll-like receptor 4.
